# The Perception of the Anesthesiologist Among the Medical, Paramedical and Administrative Staff

**DOI:** 10.3389/fmed.2022.852888

**Published:** 2022-04-21

**Authors:** Jean Selim, Marc Selim, Zoé Demailly, Thierry Wable, Thomas Clavier, Emmanuel Besnier, Bertrand Dureuil, Benoît Veber, Zoubir Djerada, Vincent Compere

**Affiliations:** ^1^Department of Anesthesiology and Critical Care, Rouen University Hospital, Rouen, France; ^2^Normandy Univ, UNIROUEN, INSERM U1096, Rouen, France; ^3^Departement of Linguistics, Faculty of Medicine, University of Rouen, Rouen, France; ^4^Department of Pharmacology, EA3801, SFR CAP-Santé, Reims University Hospital, Reims, France

**Keywords:** anesthesiologist, perception, health professionals, sociology, paramedical staff

## Abstract

**Introduction:**

Anesthesia remains a young medical discipline still relatively unknown by the general public and probably by some health professionals. The objective of the study was to evaluate the perception of anesthesiologist by health professionals working with this specialty.

**Methods:**

We distributed a computerized survey to physicians, residents, paramedical, midwives, and administrative staff in different hospitals between April and July 2018 in Normandy, France. The survey included 38 questions on 6 different topics: communicated image, skills and knowledge, communication, place in patient care, workload, and initial anesthesiologist formation. The survey was validated by a semi-directive interview methodology. A Likert scale from ×2 to +2 (“completely disagree” to “completely agree”) was used for each item.

**Results:**

Six hundred and twenty five out of 2,000 surveys sent were analyzed. The anesthesiologist conveys an image of serenity (+0.94 ± 0.79), has a high degree of responsibility (+1.72 ± 0.59) with important decision-making power (+1.39 ± 0.82). He guarantees patient safety and comfort (+1.07 ± 0.88) with his/her dual competence in anesthesia and intensive care (+1.36 ± 0.82). Anesthesiology requires teamwork (+1.68 ± 0.58) and good communication skills (+1.48 ± 0.73). The anesthesiologist is not perceived as a service provider (−0.33 ± 1.15) but is the physician responsible for perioperative care (+1.69 ± 1.00). His/her workload is moderately perceived as high (+0.71 ± 1.17) but is confronted with potentially conflictual relationships with colleagues from other specialties (+1.40 ± 0.68) and stressful situations (+1.44 ± 0.80).

**Conclusion:**

The overall perception of the anesthesiologist in our study appears to be good.

## Introduction

Anesthesia and intensive care medicine is a young medical discipline. Although it has been practiced since antiquity, no formal specialty was dedicated to this practice until 1934 when surgeons created the “Society for the Study of Anesthesia and Analgesia” in France and until 1940 when the American Board of anesthesiology separated from the American board of surgery and became an independent entity in the United States (US) (1). The number of anesthesiologists rapidly increased from 170 in 1960 to more than 10,000 in France and exceed 50,000 in the US in 2020 (2, 3). In parallel with the constant progress in this new medical specialty, the field of practice and the responsibilities of the anesthesiologist have continued to progress, generating a lack of knowledge and some confusion about the profession. Indeed, this specialty remains largely unknown to the general public and the anesthesiologist's notoriety according to the patient is low (4). Similarly, the media have a fragmented knowledge of this profession (5). In many countries, a majority of the population does not know that the anesthesiologist is a qualified doctor (6, 7). Within the French anesthesia community, the image of an anesthesiologist has greatly improved, from the “surgeon's subordinate or assistant” to a polyvalent perioperative doctor with abilities to animate Intensive Care Units, making this specialty one of the most popular among medical students choosing at the French National Ranking Examination (8, 9).

Faced with the development of acts under anesthesia, the anesthesiologist is confronted with a growing number of interlocutors, going beyond the usual field of surgery, particularly the emergence of interventional radiological or endoscopic techniques. Furthermore, with the evolution of the discipline to perioperative medicine, the anesthesiologist is increasingly confronted with non-medical personnel in the surgeries departments or during consultations (10). Finally, with the lack of anesthesiologists in France, hospital administrators have discovered the importance of the presence of anesthesiologists in the hospital (11). There is no data on the perception of anesthesiologists by other professionals working in hospital structures, whether public or private. The objective of our study was to assess the image that anesthesiologist conveys to different health professionals.

## Materials and Methods

We performed a prospective, observational, multicenter survey including one University hospital, three public general hospitals, and two private hospitals in the North-West of France. The inclusion period was from April to July 2018. The local Ethics and Evaluation Committee for Non-Interventional Research of the University Hospital approved the study (CERNI E2017-08).

### Population

The target population was all personnel who could work or interact with the anesthesiologist. This included **medical staff** (surgical or medical specialties (senior physicians or resident), **midwives**, **paramedical staff** [Certified Registered Nurse Anesthetist (CRNA), Operating Room Nurse (ORN), State Registered Nurse (SRN) in surgery service, in the Post-Anesthesia Care Unit (PACU) and, in Intensive Care Unit (ICU), Nursing Assistant (NA)] and the **administrative and managerial staff**. Staff not belonging to the above-mentioned professional categories were excluded.

### Elaboration of the Survey

**Figure 1:**
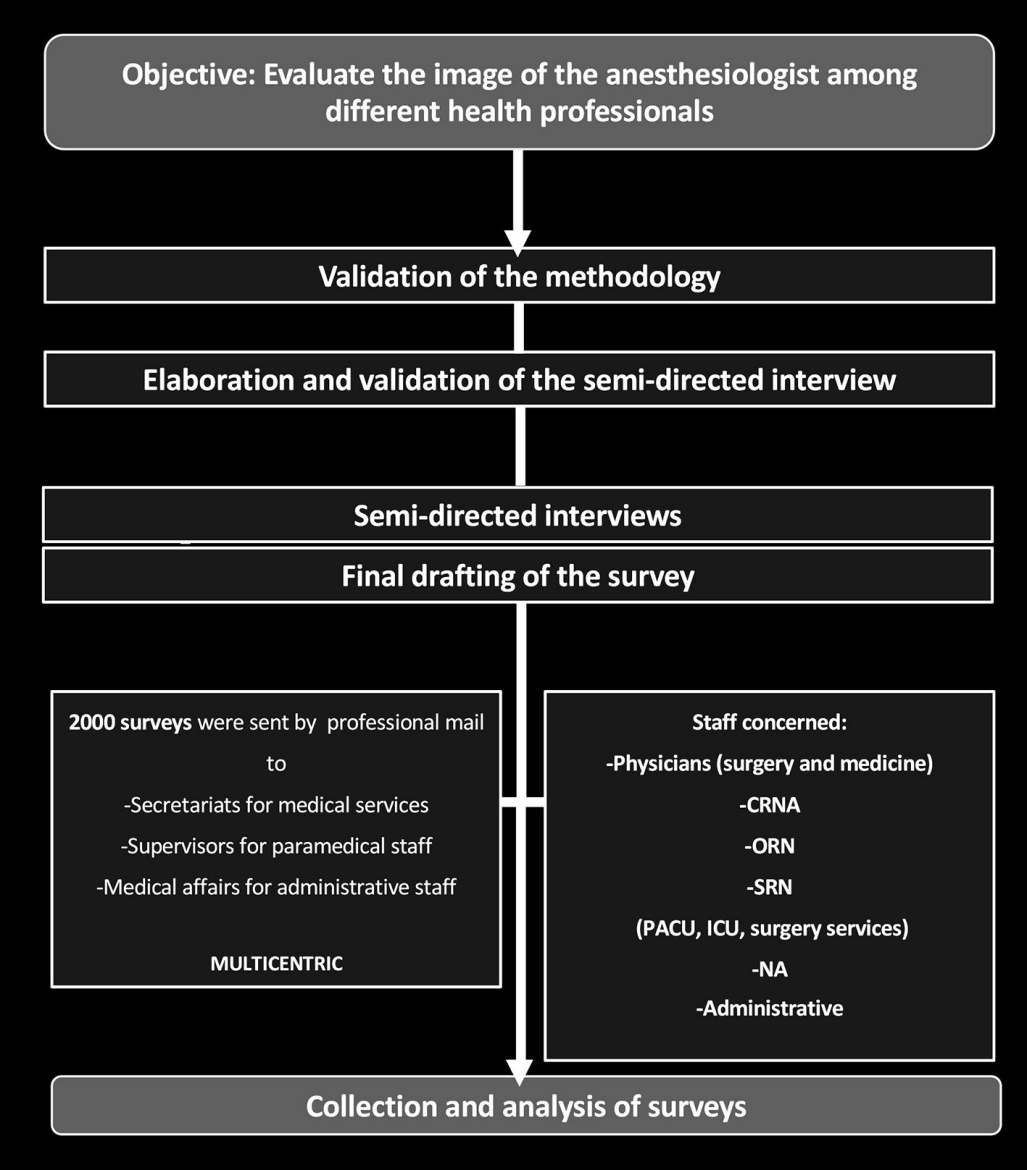
Study design. For Likert scale, data are expressed as mean ± standard deviation (SD). CRNA, Certified Registered Nurse Anesthetist; CCU, intensive Care Unit; NA, Nursing Assistant; ORN, Operating Room Nurse; PACU, Post-Anesthesia Care Unit; SRN, State Registered Nurse.

As usually recommended, we followed a three steps procedure for the elaboration of the survey (12, 13): (1) **investigation phase**, performed using the existing literature and by analyzing the semi-directive interviews; (2) **creation of the survey; (3) validation of the survey**. Each one of these steps is crucial to obtain good psychometric qualities (sensitivity, fidelity, and validity). The overall methodology used to produce the survey was validated by a University professor in psychological and social sciences. The design of the study is summarized in Figure 1.

### Step 1: Investigation Phase

After a bibliographical research phase, **semi-directive interviews** were conducted with anesthesiologists from the different hospitals (interview duration = 45 min). As the data collection was qualitative and the population concerned only anesthesiologists, a limited number of semi-directive interviews (*n* = 10) was sufficient (13, 14). This first phase provided precise and objective information on the perception of the anesthesiologist's profession and the supposed perception of other professionals. The semi-directive interviews included the general research question and the list of themes addressed (Supplementary File 1). The conduct of these semi-directive interviews was validated by a professor of communication.

### Step 2: Elaboration of the Survey

The analysis and synthesis of the answers from the interviews helped us to list the real or supposed positive and negative representations of the anesthesia profession in as exhaustive manner as possible (Supplementary File 2). We were thus able to design the final survey, which includes 38 items divided into six main themes). The first section aimed to characterize the profile of the respondent: age, gender, profession, number of years of practice, and work location. The second section consisted of 38 items aimed at assessing the importance given by the respondents to the various positive or negative, real or supposed, representations of the anesthesiologist profession. The six main themes were: **the image communicated, the skills and knowledge, the practice of anesthesia, the place in patient care, the workload, and the initial formation of the anesthesiologist**. Each item was scored using a 5 levels Likert scale ranging from “completely agree” (+2) to “completely disagree” (−2) (Supplementary File 3).

### Step 3: Validation of the Survey

We asked a group of anesthesiologists working in our institution (*n* = 10) to respond to the survey, indicating which items could be improved and if the survey tool was relevant and coherent. This final step of validation of our survey resulted in a minor reformulation of three items (items 29, 30, 31).

### Distribution of the Survey and Data Collection

The anonymized survey was distributed via a computerized version produced by Google Forms software. The weblink to the survey was distributed to all medical secretariats, paramedical supervisors, and administrative staff. According to French law, individual participant consent is not required for this study (15).

### Statistical Analysis

As this was an original study, we did not have the data to calculate the sample size. Arbitrarily, we expected to collect a minimum of 600 responses. With an estimated average response rate of 30% according to the literature on online studies, we, therefore, sent the survey to 2,000 persons (16). The use of the Likert scale comprising five levels of approval associated with a numerical value enabled us to obtain for each representation a mean evaluation (***Me***) of the entire interviewed population, allowing a hierarchization of its representations but also the comparison of the same representation in different groups of healthcare professionals. The calculation of the mean was acceptable because we were looking for general trends and we had a large sample (17–19). We then compared the different data collected between 4 groups for all survey items: a group including all physicians (group “**Med**”), a group including all paramedical staff (group “**PM**”), a group including midwives (group “**MW**”) and, a group including all administrative staff (group “**Adm**”). For *Me* analysis, the Gaussian distribution of data was assessed using the Kolmogorov–Smirnov test. We compared the data using a non-parametric Kruskal-Wallis test followed by Dunn's multiple comparison test to highlight significant differences (*P* < 0.05) between the four groups. For continuous measurements, data are presented as mean ± standard deviation (SD) for normal distribution and, median [IQR: 25th and 75th percentiles] for non-normal distribution. For qualitative parameters, data are presented as number of case (*n*), percentage (%). When a significant difference between the Med group and one of the other groups (PM, MW, or Adm group) was found in univariate analysis (*P* < 0.05), we then performed a multivariate analysis using a generalized linear model (GLM) to search for associated factors (age, sex, practice location, specialty, and the number of years of practice) positively or negatively influencing the change in mean evaluation for the physician group (Med group). The *Me* can be considered as a reliable indicator, indeed we analyzed the score data with a univariate test and a multivariate test using a generalized linear model. To test the validity of the GLM, three assumptions were checked on the residues: (1) no outliers: the minimum and maximum values of the standardized residual are within [– 3, + 3] values; (2) the data points must be independent using the Durbin–Watson test; and (3) the distribution of the standardized residuals should be normal, with mean = 0 and a constant variance not different from 1, and graphically by means of a histogram, scatterplot, Q–Q plot and normal probability plot (scatterplot of standardized residual vs. standardized predicted value or Q–Q plot), and finally by a scatterplot of observed vs. predicted value. Multicollinearity was also checked using collinearity statistics (variance inflation factor of −1) (20–23). Throughout the study, a value of *P* < 0.05 was considered significant. Statistics were performed using GraphPad Prism 8.0 and R version 3.1.4 software.

## Results

**Figure 2:**
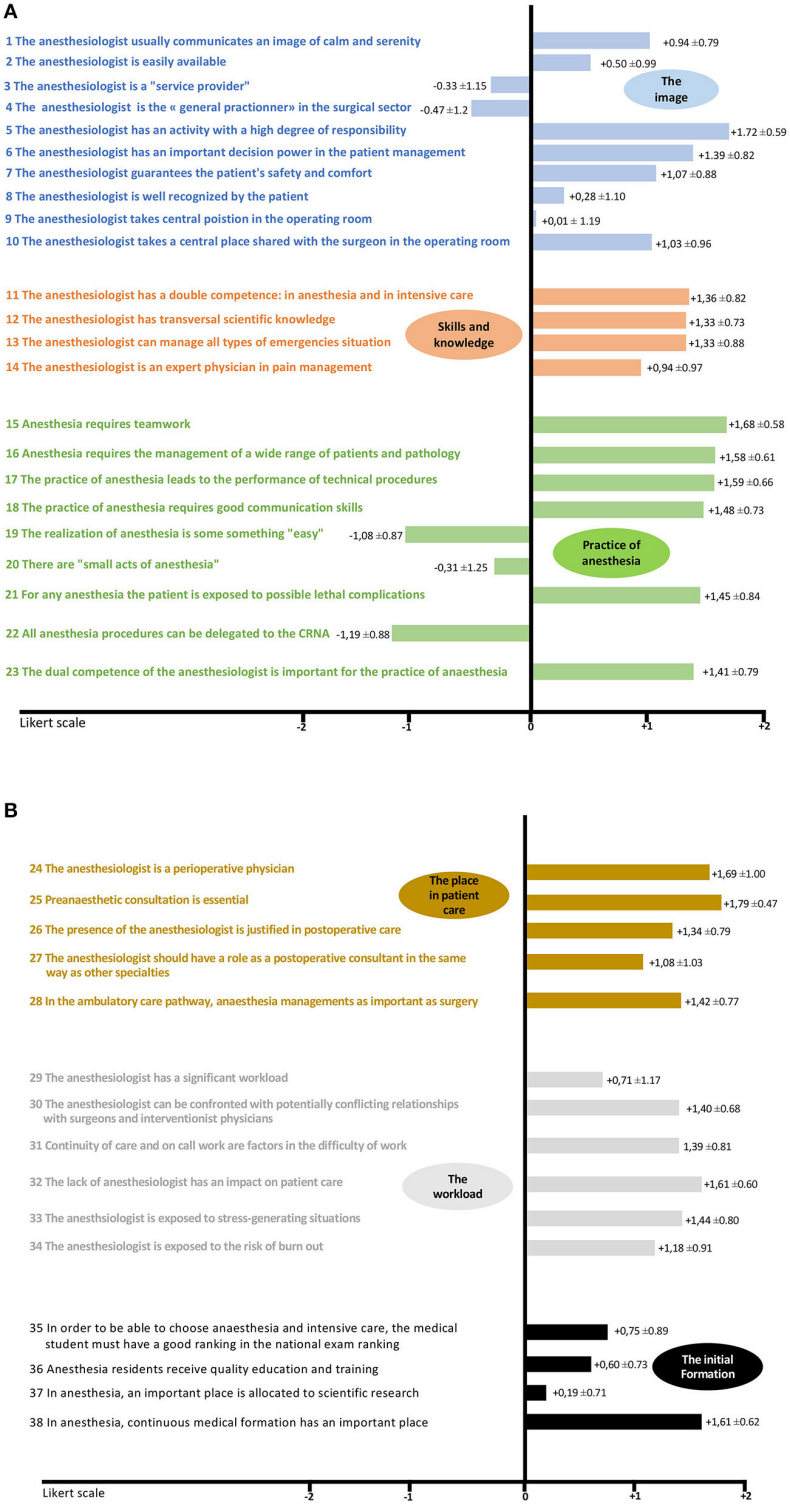
**(A)** Mean evaluation for each question of the survey on a Likert scale from −2 to +2. **(B)** Mean evaluation for each question of the survey on a Likert scale from −2 to +2.

From 22 May 2018 to 9 July 2018, we collected 629 surveys out of 2000 sent out (31% response rate). The demographic data are shown in Table 1 and the distribution between the different medical and surgical specialties in Table 2. **Analysis of the mean evaluation (*Me*) for each question is represented in** Figure 2. The analysis of the *Me* found significant differences between the Med group and PM, MW, Adm groups only for items 2, 3, 6, 8, 9, 10, 11, 14, 19, 22, 28, and 29. We have chosen to present the 5 results of the questions that seemed to us the most relevant (questions 2, 3, 8, 22, and 29).

**Table 1:** Baseline characteristics of the interviewees.

**Profession**	***n* (%)**	**Male (%)**	**Female (%)**	**Age (y)**	**Number of years of practice**	**University public hospital *n*/total number (%)**	**Public hospital *n/*total number (%)**	**Private clinics *n*/total number (%)**
Global population	629 (100)	186 (30)	443 (70)	33 [29–42]	7 [3–17]	265 (42)	339 (54)	25 (4)
Physicians Surgeons Medical speciality	288/629 (46)	135/186 (73)	153/443 (34)	31 [29–37]	4 [2–8]	164/265 (62)	103/339 (30)	21/25 (84)
Surgeons	111/629 (18)	58/186 (31)	53/443 (12)	31 [29–34]	4 [2–6]	89/265 (34)	20/339 (6)	2/25 (8)
Medical specialties	177/629 (28)	77/186 (42)	100/443 (22)	36 [29–40]	4 [2–11]	75/265 (28)	83/339 (24)	19/25 (76)
Paramedical staff CRNA ORN SRN PACU SRN CCU SRN NA	230/629 (36)	39/186 (21)	191/443 (43)	36 [28–46]	10[5–21]	81/265 (31)	145/339 (43)	4/25 (16)
CRNA	31/629 (5)	16/186 (9)	15/443 (4)	39 [35–51]	10 [7–23]	15/265 (6)	16/339 (6)	0/25 (0)
ORN	20/629 (3)	4/186 (2)	16/443 (3)	40 [32–48]	12 [6–25]	12/265 (5)	8/339 (3)	1/25 (4)
SRN in SS	111/629 (18)	6/186 (3)	105/443 (24)	34 [26–44]	10 [4–20]	23/265 (9)	88/339 (26)	3/25 (12)
SRN in PACU	14/629 (2)	4/186 (2)	10/443 (2)	37 [30–57]	15 [6–29]	8/265 (3)	6/339 (1)	0/25 (0)
SRN in ICU	20/629 (3)	5/186 (3)	15/443 (4)	26 [25–31]	7 [3–12]	14/265 (5)	6/339 (1)	0/25 (0)
NA	30/629 (5)	3/186 (2)	27/443 (6)	38 [31–61]	16 [9–24]	9/265 (3)	21/339 (6)	0/25 (0)
Midwives	46/629 (8)	2/186 (1)	44/443 (10)	31 [26–35]	7 [3–12]	12/265 (4)	34/339 (10)	0/25 (0)
Administrative and managerial staff	65/629 (10)	10/186 (5)	55/443 (13)	44 [34–61]	18 [10–32]	8/265 (3)	57/339 (17)	0/25 (0)

*Data for normal distribution are expressed as mean ± Standard Deviation. For non-normal distribution, data are expressed as median [25–75th percentiles]. For qualitative parameters, data are presented as number of patient/total number of patients (% of patients)*.

*CRNA, Certified Registered Nurse Anesthetist; SRN in SS, State Registered Nurse in surgery service; SRN in ICU, State Registered Nurse in Intensive Care Unit; SRN in PACU, State Registered Nurse in Post-Anesthesia Care Unit;ORN, Operating Room Nurse; NA, Nursing Assistant*.

**Table 2:** Repartition of the surgical and medical specialties.

**Specialties**	***n*/total number (%)**
**Global population**	**288 (100)**
**Surgical specialties**	**111/288 (38)**
Orthopedics	19/288 (6.5)
Ear nose and throat and maxillofacial surgery	15/288 (5)
Ophthalmology	6/288 (2)
Neurosurgery	4/288 (1)
Gynecology obstetrics	20/288 (7)
Digestive surgery	18/288 (6)
Cardiac surgery	1/288 (0.5)
Pediatric surgery	1/288 (0.5)
Plastic surgery	4/288 (1)
Thoracic surgery	3/288 (1)
Urologic surgery	6/288 (2)
Vascular surgery	1/288 (0.5)
Not specified	13/288 (5)
**Medical specialties**	**177/288 (62)**
Cardiology	7/288 (2.5)
Dermatology	3/288 (1)
Gastroenterology	8/288 (3)
Geriatric	7/288 (2.5)
Medical gynecology	1/288 (0.5)
Hematology	1/288 (0.5)
Infectiology and tropical disease	1/288 (0.5)
Internal medicine	7/288 (3)
General medicine	15/288 (5)
Physical Medicine and Rehabilitation	4/288 (1)
Nephrology	8/288 (3)
Biology	9/288 (3)
Neurology	4/288 (1)
Nutrition	1/288 (0.5)
Oncology	1/288 (0.5)
Pediatric	6/288 (2)
Pneumology	13/288 (5)
Psychiatry	12/288 (4)
Medical intensive care medicine	6/288 (2)
Radiology	13/288 (4)
Rheumatology	7/288 (3)
Public health	1/288 (0.5)
Emergency	21/288 (7)
Not specified	21/288 (7)

*The bold values show the difference between medical specialties and surgical specialties*.

### Question 2: Is the Anesthesiologist a Professional Available?

**Figure 3:**
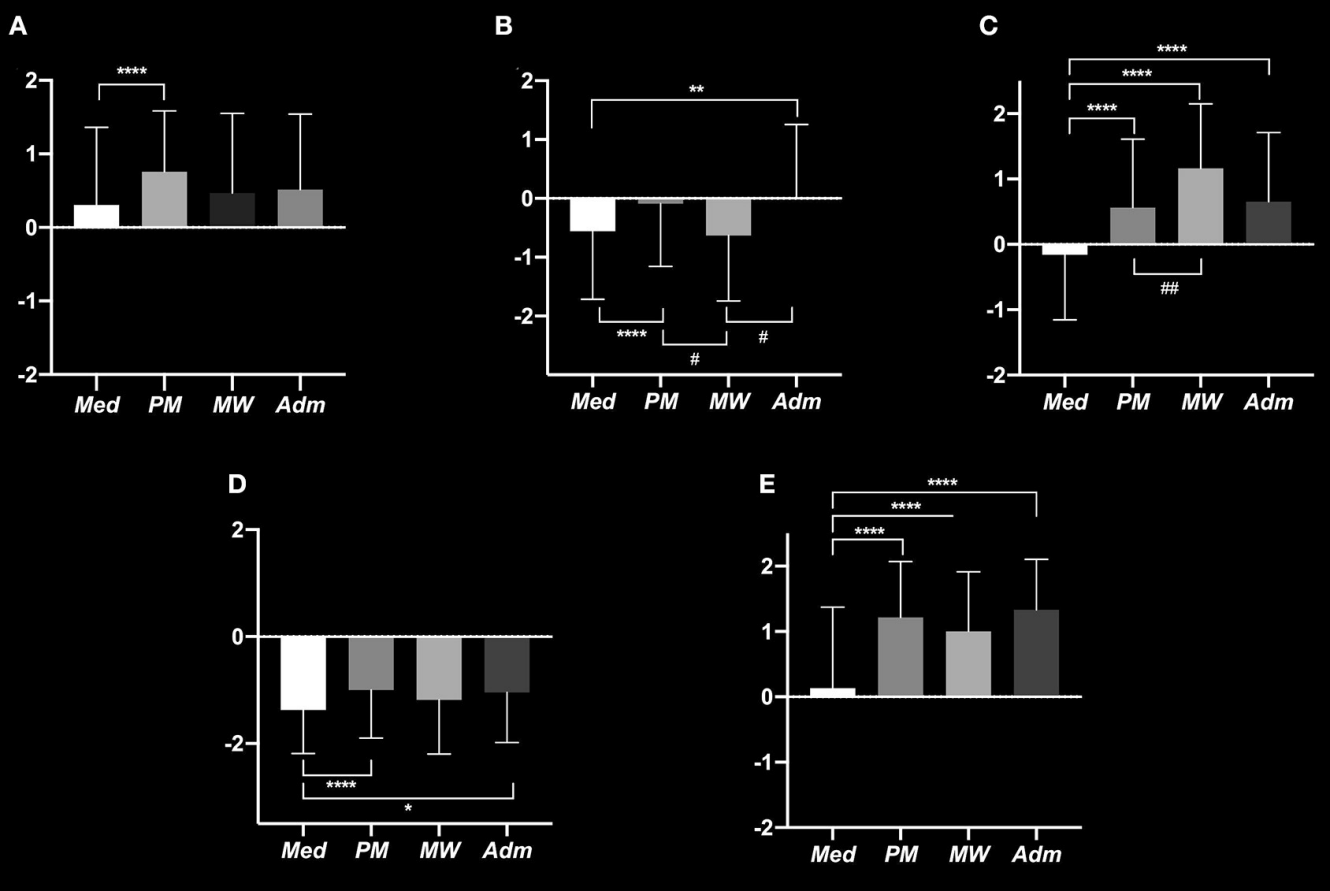
Mean Evaluation on a Likert scale from -2 to +2 for: Question 2, Is the anesthesiologist a professional available? **(A)** Question 3, Is the anesthesiologist a service provider? **(B)** Question 8, Is the anesthesiologist a professional recognized by the patient? **(C)** Question 22, All acts of anesthesia can be transferred to the certified registered nurse anesthetist? **(D)** Question 29, Does the anesthesiologist have too much workload? **(E)**. ^*^P < 0.05 vs. Med; ^**^P < 0.01 vs. Med; ^****^P < 0.0001 vs. Med; ^#^P < 0.05 vs. MW, ^*##*^P < 0.01 vs. MW. Med, Medical staff; MW, Midwives.

In univariate analysis, we observed a significantly higher *Me* in the PM group compared to the Med group (PM, +0.76 ± 0.82 vs. Med, +0.30 ±1.06, *P* < 0.0001) (Figure 3A). On Multivariate analysis, we showed that the gynecologist-obstetrician group is associated with a significant increase of the *Me* (gynecologist-obstetrician: +0.83 ±0.27, *P* = 0.003). A higher number of years of practice and practicing in public hospital are significantly associated with an increase of the *Me* (number of years of practice: +0.01 ± 0.003, *P* < 0.0001 and public hospital: +0.35 ± 0.12, *P* = 0.0063). Practicing in a University hospital significantly decreases the *Me* (University hospital: −0.25 ± 0.12, *P* = 0.039) (Table 3). Data by different categories of responders are available in Supplementary Figure 1.

**Table 3:** Multivariate analysis with associated factors positively or negatively influencing the change in mean evaluation for the Med group.

	**Number of years of practice**	**Practicing in a public hospital**	**Practicing in a University hospital**	**Visceral surgeon**	**Orthopedic surgeon**	**Surgical specialties**	**Medical specialties**	**Urological surgeon**	**Ear nose and throat surgeons**
Question 2: Is the anesthesiologist a professional available?	**+**0.01 ± 0.003 *P* < 0.0001	**+**0.35 ± 0.12 *P* = 0.0063	**–**0.25 ± 0.12 *P* = 0.039	-	-	-	-	-	-
Question 3: Is the anesthesiologist a service provider?	-	-	-	**–**1.13 ± 0.57 *P* < 0.049	**+**0.85 ± 0.28 *P* < 0.003	-	-	-	-
Question 8: Is the anesthesiologist a professional recognized by the patient?	-	**+**0.42 ± 0.12 *P* < 0.0001	**–**0.43 ± 0.11 *P* < 0.001	-	-	**–**0.40 ± 0.10 *P* < 0.0001	**+**0.31 ± 0.10 *P* < 0.01	-	-
Question 22: All acts of anesthesia can be transferred to the CRNA?	-	-	-	-	**+**2.36 ± 0.80 *P* < 0.01	-	-	-	-
Question 29: Does the anesthesiologist have too much workload?	-	**+**1.1 ± 0.1 *P* < 0.0001	**–**1.17 ± 0.13 *P* < 0.0001	**–**1.08 ± 0.35 *P* = 0.0026	-	**–**0.70 ± 0.14 *P* < 0.0001	**+**0.60 ± 0.14 *P* < 0.0001	**–**1.50 ± 0.50 *P* < 0.001	**–**0.79 ± 0.37 *P* < 0.05

*CRNA, Certified Registered Nurse Anesthetist*.

*The variation of the Mean evaluation is expressed as mean ± SD*.

### Question 3: Is the Anesthesiologist a Service Provider?

In univariate analysis, we observed a significantly higher *Me* in the PM and Adm groups compared to the Med group, (respectively PM, −0.08 ± 1.06 vs. Med, −0.55 ± 1.60, *P* < 0.0001 and Adm, 0.00 ± 1.25 vs. Med, −0.5 ± 1.16, *P* < 0.01). *Me* is significantly higher in the PM and Adm groups compared to the MW groups (PM, −0.08 ±1.06 vs. MW, −0.63 ± 1.11, *P* < 0.05 and Adm, 0.00 ± 1.25 vs. MW, –.63 ± 1.11, *P* < 0.05) (Figure 3B). Multivariate analysis showed that visceral surgeons contribute to decrease the *Me* (visceral surgeons: −1.13 ±0.57, *P* < 0.049) while orthopedic surgeons contribute to increase the *Me* (orthopedic surgeons: +0.85 ± 0.28, *P* < 0.003) (Table 3). Data by different categories of responders are available in Supplementary Figure 1.

### Question 8: Is the Anesthesiologist a Professional Recognized by the Patient?

In the univariate analysis, we observed a significantly higher *Me* in the PM, MW, and Adm groups compared to the Med group (PM, +0.56 ± 1.04 vs. Med,−0.15 ± 0.99, P <0.0001; MW, +1.16 ± 0.98 vs. Med,−0.15 ± 0.99, *p* < 0.0001; Adm, +0.65 ± 1.06 vs. Med, −0.15 ±0.99, *P* < 0.0001). We also found a significantly higher *Me* in the MW group compared to the PM group (MW, +1.16 ± 0.98 vs. PM, +0.56 ± 1.04, *P* < 0.01) (Figure 3C). In multivariate analysis, we observed a dichotomy between the different specialties in favor of a decrease of *Me* for surgeons (surgeons: −0.40 ± 0.10, *P* < 0.0001) and an increase of *Me* for medical specialties (medical specialties: +0.31 ± 0.10, *P* < 0.01). There was a significant increase of *Me* in the general public hospitals' subgroup whereas the University hospital subgroup tends to significantly decrease *Me* (public hospital: +0.42 ± 0.12, *P* < 0.0001 and University hospital: −0.43 ± 0.11, *P* < 0.001) (Table 3). Data by different categories of responders are available in Supplementary Figure 1.

### Question 22: All Acts of Anesthesia Can Be Transferred to the CRNA?

Univariate analysis found a significantly higher *Me* in the PM group compared to the Med group (PM, −1.00 ± 0.89 vs. Med, −1.37 ± 0.81, *P* < 0.0001) and a higher *Me* in the Adm group compare to the Med group (Adm, −1.04 ±0.93 vs. Med, −1.37 ±0.81, *P* < 0.05) (Figure 3D). In multivariate analysis, the orthopedic surgeon was associated with an increase of *Me* (orthopedic surgeons: +2.36 ± 0.80, *P* < 0.01) (Table 3). Data by different categories of responders are available in Supplementary Figure 1.

### Question 29: Does the Anesthesiologist Have Too Much Workload?

Univariate analysis showed a significant increase of the *Me* in the PM, MW, and Adm groups compared to the Med group (PM, +1.21 ± 0.85 vs. Med, +0.13 ± 1.24, *P* < 0.0001; MW, +1.00 ± 0.91 vs. Med, +0.13 ± 1.24, *P* < 0.0001 and Adm, +1.33 ± 0.77 vs. Med, +0.13 ±1.24, *P* < 0.0001) (Figure 3E).

Multivariate analysis showed that the surgeons' response is associated with a significant decrease of the *Me* (surgeon: −0.70 ± 0.14, *P* < 0.0001) with a significant decrease for visceral surgeons (visceral surgeon: −1.08 ± 0.35, *P* = 0.0026), for urological surgeons (urological surgeon: −1.50 ± 0.50, *P* < 0.001) and for ear nose and throat (ENT) surgeons (ENT surgeons: −0.79 ± 0.37, *P* < 0.05). The response of medical specialties significantly increases the *Me* (medical specialties: +0.60 ± 0.14, *P* < 0.0001) as well as working in a public hospital (public hospital: +1.1 ± 0.1, *P* < 0.0001). The fact of working in a University hospital is associated with a significant decrease of the *Me* (University hospital: −1.17 ± 0.13, *P* < 0.0001) (Table 3). Data by different categories of responders are available in Supplementary Figure 1.

## Discussion

### The Image Promoted by the Anesthesiologist

We observed that the anesthesiologist conveys a positive image: he/she inspires calmness and serenity, has a high degree of responsibility, benefits from significant decision-making power (especially from the point of view of the paramedical staff and in particular for the CRNA) and he/she is responsible for the patient's safety and comfort. These last two elements have already been shown to be shared by the general public (24). Other points remain unclear in the eyes of those interviewed, notably the “service provider aspect” that can potentially result from the practice of anesthesiology, characterized by the punctual nature of its acts and by the possible interchangeability of the anesthesiologists between them. This vision of the “service provider” in the study by Peyrache *et al*. appeared to be the second element that may make medical students hesitate in their choice of anesthesiology at the end of the course (the activity with a high degree of responsibility being the first) (25). In our study, it seems that the paramedical staff most share this vision of provider. This can be explained in part by the point of view of the ORN. They only participate in intraoperative anesthetic management and are often not involved in the pre-and post-operative period or the intensive care activity. In our multivariate analysis, visceral surgeons, unlike orthopedic surgeons, were more opposed to this reductive image of the profession. This discrepancy can probably be explained by a better perception of the challenges of anesthetic management on the part of visceral surgeons. Indeed, visceral surgeons manage a greater proportion of patients with multiple comorbidities and major surgeries, often requiring postoperative monitoring in the intensive care unit.

The anesthesiologist seems to be more available to paramedical staff than to medical staff. The paramedical staff is often the main interlocutors of the anesthesiologist, especially in the wards, during pre-anesthetic or post-operative visits both in the surgery department or intensive care unit. Although the anesthesiologist has multiple knowledge and skills, he/she is not qualified as a “general practitioner in the surgical sector” by all health professionals (8). Several studies have highlighted the patient's lack of knowledge of the anesthesiology profession (26–28). Our study shows that it is the doctors, in front of the paramedical staff, who are the most aware of it, and in particular the surgeons (probably because they make the observation directly with the patient), unlike the medical specialties.

### The Skills and Knowledge of the Anesthesiologist

The diversity of the exercise of anesthesia and intensive care appears to be well-recognized. The anesthesiologist is identified as a doctor with a wide transversal scientific knowledge, able of treating all types of emergencies, and he/she is well-recognized for his dual competence in anesthesia and intensive care. However, it appears to be the paramedics who are most aware of this, and in particular the CNAR. This perception is surprisingly worse for the management of analgesia (*Me* = 0.94), even though this is a major challenge in anesthesia management. The paramedical staff again seem to be more aware of this than the doctors (except for obstetrician-gynecologists). Indeed, paramedics often directly request the anesthesiologist for the management of postoperative analgesia in surgical departments for example. It is the midwives who have the best perception of this competence and it is reasonable to think that this is partly linked to the specific role of the anesthesiologist in obstetric analgesia, in particular the performance of epidural anesthesia (29).

### The Constraints of the Profession

Anesthesia and intensive care are identified as stressful activities, with exhausting on-call work, exposing to the risk of burnout (30). This last problem is identified within the profession itself and is the subject of much attention (31). Indeed, anesthesia is certainly one of the most stressful specialties. The anesthesiologist is daily exposed to stressful situations such as the management of life-threatening scenarios or end life decisions. In addition, this specialty includes night shifts as well as festivities and weekends. It is therefore not surprising to find a high incidence of burnout in this specialty. The potentially conflicting relationships with surgeons and interventional physicians are also recognized by all health professionals. This almost “historical” conflictual relationship of conflict has even been the subject of recommendations issued by the high authority for health in 2015 in France (32, 33). This point of view is mainly expressed by physicians, especially surgeons, and not by paramedical or even administrative staff. This can be explained by a lack of knowledge of the activity of the anesthesiologist outside the operating room (post-operative visits, management of PACU and intensive care units) (8).

### Limits

This survey mainly contained closed questions. This limitation of answers allowed us to achieve certain conciseness, but also to perform a quantitative analysis of the data. However, we were exposed to a loss of information and nuance compared to what could be obtained with open answers or interviews. Moreover, the order of the questions was arbitrarily defined and grouped into sub-groups, which exposes us to the phenomenon of anchoring (the answer to one question can influence the answer to another, the respondent wants to keep coherence even unconsciously), a bias that is difficult to avoid and which is difficult to evaluate in our study. In addition, the way some questions are formulated may induce a confirmation bias. In this case, the question could positively or negatively confirm a preconceived idea. Our population also includes an important part of young physicians and paramedics working mainly in public hospitals. Our population is, therefore, not wholly representative of a substantial proportion of medical and paramedical professionals. Finally, our study reflects the French vision of the anesthesiologist with its history, its current difficulties (such as the shortage), and its specificities (such as the dual competence in anesthesia and intensive care). This vision is probably different in other countries even if common points probably exist.

## Conclusion

This study is the first to describe the perception of the anesthesiologist by his different interlocutors. The overall perception is quite good even if there are interprofessional variations. This image contrasts with the self-depreciation generally perceived by anesthesiologists. With the current pandemic, the anesthesiologist plays a key role in the management of patients and the coordination of the different hospital actors highlighting the anesthesiologist profession. It would be interesting to repeat this study in the post-pandemic period.

## Data Availability Statement

The data underlying this article will be shared on reasonable request by the corresponding author.

## Ethics Statement

The studies involving human participants were reviewed and approved by the Local Ethics and Evaluation Committee for Non-Interventional Research of the University Hospital approved the study (CERNI E2017-08). Written informed consent for participation was not required for this study in accordance with the national legislation and the institutional requirements.

## Author Contributions

JS and MS were involved in the study conception and design, in the acquisition of data, in statistical analysis, in analysis and interpretation of data, and in manuscript draft. ZD was involved in the study conception and design, in the acquisition of data, in analysis and interpretation of data, and manuscript draft. BD and BV were involved in manuscript revision. TW, EB, TC, and ZD were involved in the study conception and design and manuscript revision. VC was involved in the study conception and design, in resident recruitment, in study coordination, in the interpretation of data, and in manuscript revision. All authors contributed to the article and approved the submitted version.

## Funding

Support was provided solely from departmental sources.

## Conflict of Interest

The authors declare that the research was conducted in the absence of any commercial or financial relationships that could be construed as a potential conflict of interest.

## Publisher's Note

All claims expressed in this article are solely those of the authors and do not necessarily represent those of their affiliated organizations, or those of the publisher, the editors and the reviewers. Any product that may be evaluated in this article, or claim that may be made by its manufacturer, is not guaranteed or endorsed by the publisher.

## Supplementary Material

Supplementary File 1 Semi-directive interviews.

Supplementary File 2 Summary of interviews: only responses to questions with more than 60% of matching answers have been listed (*n* = 10).

Supplementary File 3 Survey of the study.

Supplementary Figure 1 Mean evaluation on a Likert scale from −2 to +2 by categories of responders. Question 2, Is the anesthesiologist a professional available? **(A)** Question 3, Is the anesthesiologist a service provider? **(B)** Question 8, Is the anesthesiologist a professional recognized by the patient? **(C)** Question 22, All acts of anesthesia can be transferred to the certified registered nurse anesthetist? **(D)** Question 29, Does the anesthesiologist have too much workload? **(E)**. ^*^*P* < 0.05 vs. Med; ^**^*P* < 0.01 vs. Med; ^****^*P* < 0.0001 vs. Med; ^#^*P* < 0.05 vs. MW; †*P* < 0.05 vs. ORN; ††††*P* < 0.0001 vs. ORN. Adm, administrative and managerial staff; CRNA, Certified Registered Nurse Anesthetist; Med, Medical staff; MW, Midwives; NA; Nursing Assistant; ORN, Operating Room Nurse; SRN, State Registered Nurse; SRN-PACU, State Registered Nurse in the Post-Anesthesia Care Unit; SRN-ICU, State Registered Nurse in Intensive Care Unit.
