# Supplementary material for: The Perception of the Anesthesiologist Among the Medical, Paramedical and Administrative Staff
Source: Front Med (Lausanne). 2022 Apr 21;9:852888. doi: 10.3389/fmed.2022.852888 (PMC9069072; doi:10.3389/fmed.2022.852888)
Supplement: Supplementary File 1 — Semi-directive interviews. [file Data_Sheet_1.pdf]

Supplementary File 1: semi-directive interviews

1. Interviewee Information:

-Age:...

-Gender:...

-Profession (specialty): ....

-Status (resident,senor):...

-Number of years in practice: .... -Work location: ....

2. The general research question:

-How would you characterize in one word, the perception of the anesthesiologist by other health professionals? (by perception, we mean the image, representativeness, and knowledge of the anesthesiologist function)

-Open answer: ……………………………

-Relaunch if not expressed with directed questions:

-Overall, is the perception good or bad? ……………………….

-Is this perception the same for all health professionals? Yes/No……………………….

Relaunch:

2.1 Perception by other physicians

2.1.1 The perception by surgeons

-Is there a difference between the different surgical specialties? Is there a difference between surgeries closely related to intensive care? (e.g. cardiac surgery...) vs. other surgeries?

2.1.2 Among the other specialties

2.1.2.1 The perception by interventional radiologists (including cardiologists)

2.1.2.2 The perception by endoscopic physicians (pulmonologists/gastroenterologists)

-Which medical specialty do you think has the worst perception?

Among physicians? …………………………………..

-In your opinion, has the perception of the anesthesiologist changed over the generations?

In which direction? …………………………………..

2.1.2.3 The perception by midwives

2.1.2.4 The perception by paramedical staff (Certified Registered Nurse Anesthetist (CRNA), Operating Room Nurse (ORN), State Registered Nurse (SRN)) :

2.1.2.5 The perception by the hospital administration

Does this positive or negative perception have consequences:

-On your daily practice? ………………………………..

-On patient care? ………………………………..

3. The different themes discussed on the perception of the profession

3.1 Why did you choose the profession of anesthesiologist?

-Open answer: …………………………..

-Relaunch if not expressed with directed questions :

-Was it a choice by vocation, desire, the prestige of the job? Other? ……………………………..

-Were there any physicians in the family circle? ……………………………..

-Did you have an image of the anesthesiologist before making your choice?

-If yes, which one? ……………………………..

-Does this initial perception persist? ……………………………..

3.2 What is for you the place of the anesthesiologist in the patient's care pathway?

-Open answer: ……………………………

-Relaunch if not expressed with directed questions :

-For you, the anesthesiologist is the physician of the :

Perioperative/preoperative/intraoperative/postoperative care……………………………..

-Do you think that the anesthesiologist is at the core of the patient's care? ……………………..

- In your opinion, at what time of the patient's management is the place of the anesthesiologist most misperceived? (preoperative, intraoperative, or postoperative step)

-Do you think that the contribution of the anesthesiologist in the management of the patient is well perceived by the other professionals? Yes/no……………………..

-In your opinion, does anesthetic management in the eyes of other health professionals seem to be passive or active? ……………………..

3.3 What can you say about the place of the anesthesiologist in the operating room?

-Open answer: ……………………………

-Relaunch if not expressed with directed questions :

-In your opinion, who is the main actor in the organization of the operating room? The surgeon? The anesthesiologist? Both? …………………….. Why or why not? ……………………..

-Who do you consider to be the professional responsible for the patient in the operating room? The surgeon? The anesthesiologist? Both? ……………………..

-Do you feel you are considered equal to the surgeon? To be a subordinate of the surgeon? Being a service provider? ……………………..

-Is there a place for discussion of the surgical indication? Of the surgical technique? Of the

anesthesia technique? …………………….

3.4 The place of the anesthesiologist in the postoperative period

-Open answer: ……….…………………

-Relaunch if not expressed with directed questions:

-What is (are) for you, the main axis(s) of the postoperative management under the responsibility of the anesthesiologist:

- Pain
- Management of chronic treatments
- Monitoring of chronic disease decompensation
- Monitoring of postoperative complications

-Is the role of the anesthesiologist in the early rehabilitation of the patient is more important/as important/less important than the surgeon? ……………….…………………..

-Is the role of the anesthesiologist in ambulatory care is more/as important/less important than the surgeon? ……………….…………………..

-Do you establish your postoperative prescriptions jointly with the surgeon? Yes/no

-In your opinion, who is responsible for the patient in the surgery service? The anesthesiologist or the surgeon? How would you describe the communication between the anesthesiologist and the surgeon during this specific time of care? ……………….…………………

3.5 Stress and professional practice

-Open answer: ……….…………………

-Relaunch if not expressed with directed questions:

-Is stress part of your daily practice? ………….……………………

-What is its intensity? ………….……………………

-What are the situations or elements that generate stress? ……………..………………….

-Do you feel the pressure of responsibilities? ……………..………………….

-Does it have an impact on your personal life? ……………..………………….

-Do you feel that the anesthesiologist's profession is more exposed to the risk of burn-out than other specialties? ……………..………………….

3.6 Scientific knowledge and the practice of the profession

-Open answer: …………..……………….

-Relaunch if not expressed with directed questions:

-Does the practice of anesthesia requires global knowledge of:

-Pathologies and their complications?

-Therapeutics?

-Surgical procedures? (the different times, complications...)

-Is it necessary to have an interest in technology and science? …………..……………….

-Because of his knowledge, can the anesthesiologist be or is he considered to be the hospital's "general practitioner"?…………..……………….

-Do you feel the need to constantly update your knowledge? …………..……………….

-Do you consider the place of research in anesthesia and resuscitation to be important?

-Does it seem more active than in other medical specialties? …………..……………….

-Do you think that other health care professionals optimally use the expertise of the anesthesiologist? Yes/no …………..……………….

-Does the practice of anesthesia require in certain cases an "over-specialization"?

Example: Pediatrics/obstetrics/cardiac surgery/neurosurgery

3.7 What qualities are necessary to be an anesthesiologist?

-Open answer: …………………………..

-Relaunch if not expressed with directed questions:

-Versatility/anticipation/communication...?

-In your opinion, is there a particular personality profile? …………………………..

-What is the place of technical skills in daily life? …………………………..

-Does the exercise of the profession require relational qualities? …………………………..

Examples: …………………………..

3.8 Teamwork

-Open answer: …………………………..

-Relaunch if not expressed with directed questions:

-Do you feel that the anesthesiologist work as a team? …………………………..

-Do you feel that you work as a team with the surgeon? …………………………..

-Do you feel that you are part of a multidisciplinary team? …………………………..

-Do you feel that the interchangeability of the anesthesiologists (for consultation, anesthesia, and postoperative care) is well perceived by the other professionals? ……………

-In your opinion, are the competencies or possible delegations to the CRNA identified by the other health professionals? …………………….

-How would you describe the relationship with the surgeon? Good/bad……………….

-Can the anesthesiologist be subjected to pressure from other physicians? …………………….

-How would you rate the communication between the anesthesia and surgery teams? Good /bad…………….

-How would you rate the relationship with other medical specialists? Good/bad…………….

-Example of obstetrics: midwife/obstetrician……………

-Example of endoscopists……………

-Example of radio-interventionists……………

-In your opinion, what is the main source of conflict between anesthesiologists and other health professionals? …………..

-What is the impact of the presence of the anesthesiologist in surgical staff meetings? ……

-Do you feel that the issues of anesthetic management of the patient are identified by other health professionals? If not, which ones in particular?..............................

3.9 Work time

-Open answer: …………………………..

-Relaunch if not expressed with directed questions:

-Specify the distribution of your work time: ……………………….

-Time in the operating room/time outside the operating room (visits/consultations)

-How do you estimate your workload? ……………………….

-Insufficient …………………..

-Correct…………………..

-Important …………………..

-How would you qualify the on-call duty? ……………………….

-Do you feel the impact of on-call duty on your personal and professional workload?

-Do you feel the national shortage of anesthesiologists has an impact on your workload?

-Do you think your workload is greater than that of other medical specialties? ………….

-Do you feel that your work time is well perceived? ……………………….

under or overestimated? Examples: ……………………..

3.10 Patients

-Open answer: …………………………..

-Relaunch if not expressed with directed questions:

-How would you rate patients' knowledge of the anesthesiologist profession: good or bad? -

-How would you rate patients' recognition of the anesthesiologist: good or bad? ……………..

-Is the relationship with the patient important to you? Yes/no…………………

-Do you feel medico-legal pressure from the patients? …………………

-In your opinion, which healthcare professional contributes the most to risk management and patient safety in the care process? …………………

-Do you see a difference between the surgeon/patient and anesthesiologist/patient relationship? Yes/no …………………

Elements explaining this difference in your opinion? ………………

3.11 How would you describe your work environment?

-Open answer: …………………………..

-Relaunch if not expressed with directed questions:

-How would you rate the overall work environment?

Good/bad………………

Explanatory elements? ………………

Anesthesiologist and administration:

-Do you perceive any pressure from the administrative system?

Examples: ……………

-Do you feel recognition from the administrative institution?

Examples: …………….

Anesthesia residents

-How would you rate the supervision and teaching of residents in the residency program?

Do you feel it is superior to other specialties? …………….

3.12 Intensive care

-Open answer: …………..………………

-Relaunch if not expressed with directed questions:

-What is the place of intensive care in the daily life of the anesthesiologist? …………….

-In your opinion, is it possible to dissociate the dual competence of anesthesia and intensive care? …………….

-In your opinion, are other professionals sufficiently aware of this dual competence?

-What do you think about the place of the anesthesiologist in pre-hospital emergency medicine? …………………………..

3.13 What is your overall satisfaction with your professional practice?

-Open answer: …………..………………

-Relaunch if not expressed with directed questions:

-How would you rate your quality of life? ………………………..

-Do you feel it is better than other physicians? ………………………..

-Do you feel that your practice is useful? ………………………..

3.14 The future of the specialty

-Open answer: …………..………………

-Relaunch if not expressed with directed questions:

-How do you think the perception of the anesthesiologist will change with other health professionals? …………………….

-How do you rate the overall attractiveness of the specialty? …………………….

-Would you advise students to choose this specialty? Yes/no, on what arguments?

-In your opinion, can the anesthesiologists have a legitimate place in the administrative management of healthcare institutions? …………………….

-How can we improve the general perception of the profession? …………………….

-Are you worried about the future of the specialty? …………………….

-About the possible transfer of skills to other medical disciplines: example of sedation performed by gastroenterologists

- About the possible transfer of tasks to other health professionals (anesthesiologist /CRNA)

-Do you think that anesthetic procedures are becoming commonplace?

-On the part of the anesthesiologists?

-From other health professionals?

3.15 Free comments

Abbreviations: CRNA, Certified Registered Nurse Anesthetist; ORN, Operating Room Nurse; SRN, State Registered Nurse.
