# Supplementary material for: The Perception of the Anesthesiologist Among the Medical, Paramedical and Administrative Staff
Source: Front Med (Lausanne). 2022 Apr 21;9:852888. doi: 10.3389/fmed.2022.852888 (PMC9069072; doi:10.3389/fmed.2022.852888)
Supplement: Supplementary File 2 — Summary of interviews: only responses to questions with more than 60% of matching answers have been listed (n = 10). [file Data_Sheet_2.pdf]

Supplementary File 2

Summary of interviews: only responses to questions with more than 60% of matching answers have been listed (n=10)

Synthesis of Semi-structured Interviews	
**Questions**	**Answers**
**The general research question**	
How would you characterize in one word, the perception of the anesthesiologist by other health professionals? (by perception, we mean the image, representativeness, and knowledge of the anesthesiologist function)	Spontaneous answer: mostly negative image ("bad, the status of the anesthesiologist is not valorized, he is a service provider" "perception is skewed")
Overall, is the perception good or bad?	Bad for 80% of the interviewees
Is this perception the same for all health professionals?	No for 100% of the interviewees
The perception by midwives	Good for 60% of the interviewees
The perception by paramedical staff	CRNA: Good for 100% of the interviewees

	ORN: Good for 40% of the interviewees<br>SRN: Good 70% of the interviewees
The perception by the hospital administration	Bad for 100% of the interviewees
**Why did you choose the profession of anesthesiologist?**	
Open answer: Polyvalence mentioned 6 times. Intensive care mentioned 5 times	
Did you have an image of the anesthesiologist before making your choice?	Serenity mentioned 4 times<br>The notion of multi-skills mentioned 6 times
**What is for you the place of the anesthesiologist in the patient's care pathway?**	
Spontaneous answer: Important for 90% of the interviewees	
Do you think that the anesthesiologist is at the core of the patient's care?	Yes for 100% of the interviewees
In your opinion, at what time of the patient's management is the place of the anesthesiologist most misperceived? (preoperative, intraoperative, or postoperative step)	Preoperative for 60% of the interviewees<br>Postoperative for 50% of the interviewees

Do you think that the contribution of the anesthesiologist in the management of the patient is well perceived by the other professionals?	No for 100% of the interviewees
**What can you say about the place of the anesthesiologist in the operating room?**	
Spontaneous answer: central for 70% of the interviewees	
In your opinion, who is the main actor in the organization of the operating room: the surgeon? The anesthesiologist? Both?	Both for 80% of the interviewees<br>The anesthesiologist for 20% of the interviewees
Who do you consider to be the professional responsible for the patient in the operating room?	Both for 100% of the interviewees
Do you feel you are considered equal to the surgeon? To be a subordinate of the surgeon? Being a service provider?	equal to the surgeon: no for 70% of the interviewees<br>Being a service provider: yes for 60% of the interviewees
**The place of the anesthesiologist in the postoperative period**	
What is (are) for you, the main axis(s) of the postoperative management under the responsibility of the anesthesiologist	Pain/management of chronic treatments/monitoring of chronic disease decompensation/monitoring of postoperative complications

	The 4 items have been mentioned spontaneously by 100% of the interviewees
Is the role of the anesthesiologist in ambulatory care is more important/as important/less important than the surgeon?	As important for 100% of the interviewees.
**Stress and professional practice**	
Is stress part of your daily practice?	No for 70% of the interviewees
Do you feel the pressure of responsibilities?	Yes for 100% of the interviewees
Do you feel that the anesthesiologist's profession is more exposed to the risk of burn-out than other specialties?	Yes for 70% of the interviewees
**Scientific knowledge and the practice of the profession**	
Spontaneous answer: important for 100% of the interviewees	
Can the anesthesiologist be or is he considered to be the hospital's "general practitioner"	Yes for 60% of the interviewees
Do you feel the need to constantly update your knowledge?	Yes for 100% of the interviewees

Do you consider the place of research in anesthesia and intensive care important?	Yes for 60% of the interviewees
Do you think that other health care professionals optimally use the expertise of the anesthesiologist?	No for 90% of the interviewees
**What qualities are necessary to be an anesthesiologist?**	
Spontaneous answer: "calm" cited 6 times, "serenity" cited 6 times, "ability to work in a team” cited 6 times	
What is the place of technical skills in daily life?	Important for 100% of the interviewees
Does the exercise of the profession require relational qualities?	Yes for 100% of the interviewees
**Teamwork**	
In your opinion, are the competencies or possible delegations to the CRNA identified by the other health professionals?	No for 60% of the interviewees
How would you describe the relationship with the surgeon?	Important for 100% of the interviewees
In your opinion, what is the main source of conflict between anesthesiologists and other health professionals?	A lack of communication for 60% of the interviewees.

Do you feel that the issues of anesthetic management of the patient are identified by other health professionals? If not, which ones in particular?	No for 100% of the interviewees<br>There is no specific period in particular
**Work time**	
How do you estimate your workload?	Correct for 70% of the interviewees
How would you qualify the on-call duty?	Difficult for 100% of the interviewees
Do you feel the national shortage of anesthesiologists has an impact on your workload?	Yes for 80% of the interviewees
Do you think your workload is greater than that of other medical specialties?	No for 60% of the interviewees
**Patients**	
How would you rate patients' knowledge of the anesthesiologist profession: good or bad?	Bad for 90% of the interviewees
In your opinion, which healthcare professional contributes the most to risk management and patient safety in the care process?	The anesthesiologist for 100% of the interviewees

Do you see a difference between the surgeon/patient and anesthesiologist/patient relationship?	Yes for 100% of the interviewees
**Intensive care**	
In your opinion, is it possible to dissociate the dual competence of anesthesia and intensive care?	No for 100% of the interviewees
In your opinion, are other professionals sufficiently aware of this dual competence?	Yes for 60% of the interviewees
**What is your overall satisfaction with your professional practice?**	
Spontaneous answer: good for 80% of the interviewees	
**The future of the specialty**	
How do you think the perception of the anesthesiologist will change with other health professionals?	Better for 60% of the interviewees
How do you rate the overall attractiveness of the specialty?	Good for 70% of the interviewees
How can we improve the general perception of the profession?	Better communication for 60% of the interviewees
Are you worried about the future of the specialty?	No for 90% of the interviewees

Are you worried about the possible transfer of tasks to other health professionals (anesthesiologist →CRNA)	No for 100% of the interviewees
Do you think that anesthetic procedures are becoming commonplace?	from the anesthesiologists: yes for 50% of the interviewees<br>from the other health professionals: yes for 100% of the interviewees

Abbreviations: CRNA, certified registered nurse anesthetist, ORN, operating room nurse; SRN, state register nurse.
